# In Situ Investigation of Upper Airway Occlusion in Sleep Disordered Breathing Using Ultrasonic Transducer Arrays

**DOI:** 10.3390/bios13010121

**Published:** 2023-01-10

**Authors:** Mohammad Al-Abed, Donald Watenpaugh, Khosrow Behbehani

**Affiliations:** 1 College of Engineering and Technology, American University of the Middle East, Egaila 54200, Kuwait; 2 Faculty of Engineering, The Hashemite University, Zarqa 13133, Jordan; 3 College of Engineering, The University of Texas at Arlington, Arlington, TX 76019, USA

**Keywords:** biological system modeling, biomedical ultrasound, obstructive sleep apnea

## Abstract

This work presents a novel application of ultrasound for the real-time, non-invasive investigation of occlusion of the upper airway during events of obstructive sleep apnea/hypopnea syndrome. It is hypothesized that ultrasonic pulses applied to the neck during apneic events produce spectral and temporal features that can detect apnea occurrence. Theoretical models of ultrasound propagation and an in vitro test were conducted to test this hypothesis in both transmission and reflection modes. Complete specifications and technical details of the system design and fabrication, which is mounted on each subject’s neck, are presented, including the methodology. Nine patients (seven male and two female, mean age of 42 years, with a range of 25 to 56 years, and body mass index 37.6 ± 6.6 kg/m^2^) were recruited for a full night study, which included simultaneous nocturnal polysomnography for the validation of the results. Nine temporal features and four spectral features were extracted from the envelope of the received pulse waveform. These were used to compute 26 metrics to quantify the changes in the ultrasonic waveforms between normal breathing and apneic events. The statistical analysis of the collected ultrasonic data showed that at least two or more of the proposed features could detect apneic events in all subjects. The findings establish the feasibility of the proposed method as a cost-effective and non-invasive OSAHS screening tool.

## 1. Introduction

Obstructive sleep apnea/hypopnea syndrome (OSAHS) is classified as a sleep disorder (G47.3) under the ICD-10 (International Statistical Classification of Diseases, 10th Revision), which is a subgroup of the episodic and paroxysmal disorders, which is, in turn, a group of the diseases of the nervous system [1]. It is sleep-disordered breathing (SDB), defined in adults as the cessation of breathing (apnea) or 50% or more partial airway occlusion (hypopnea) for 10 s or more and for 5 or more episodes per hour [2]. By definition, the patient continues the respiratory effort and movements during these events, with a cyclic recurrence of these episodes during the night [3,4,5]. The length of each episode is typically 20–40 s, with some rare severe cases extending beyond 60–90 s [3]. Snoring is often associated with the disorder, along with cyanosis and whole-body movements occurring at arousal [3].

Patients diagnosed with OSAHS have a high incidence of obesity, where about 70% of OSAHS patients are obese, and 40–90% of individuals with severe obesity (body mass index (BMI) > 40kg/m^2^) are apneic [6,7,8]. Obese patients have higher adipose tissue deposits in the neck, physically narrowing the airway.

There are cases where the central respiratory centers fail to increase the muscle tone of the diaphragm, and without an upper airway occlusion, a cessation of the ventilatory effort occurs, resulting in blood oxygenation desaturation and cortical arousal. These cases are defined as central sleep apneas (CSA). Mixed apnea is when a respiratory event starts with central apnea, followed by the occlusion of the airway in the concourse with a resumption of ventilatory effort [3].

Even with the increased use of home sleep testing (HST) in the last few years, nocturnal polysomnography (NPSG) remains the golden standard used for the diagnosis of OSAHS [9,10,11]. However, NPSG is costly because it involves multiple instruments, multiple night studies, labor costs associated with patient instrumentation, observation, and review of records [12].

Due to the prevalence of SDB, its socioeconomic cost, the limited number of credited sleep labs, and the high cost of a diagnostics sleep study (USD 1500–2000 per night), inexpensive and reliable SDB screening methods are highly sought after [11,12,13,14].

The underlying causes of OSAHS may differ for different patients and are related to the upper airway anatomy, obesity, airway muscular and neurological activity, arousal threshold, chemo-reflex sensitivity, and complex mechanisms that contribute to all or some of these underlying causes [14,15,16].

An anatomically small pharyngeal airway is one of the major underlying causes of OSAHS [2,14,15,16]. This is due to abnormalities in the skull bone structure and mandible location compared to the maxilla and the hard palate [2,5]. In addition, other soft tissue abnormalities, such as an increase in the volume of the tongue, the soft palate, the parapharyngeal fat pads (especially in cases of obesity), and the lateral walls surrounding the pharynx, are associated with cases of OSAHS [2,5,14]. However, anatomical abnormality only constitutes one-third of all cases of OSAHS [2].

The upper airway is kept patent (open) by the activity of the upper airway dilator muscles [2,16]: levator veli palatine, tensor veli palatine, palatoglossus, palatopharyngeus, and the genioglossus. The activation of these muscles is thought to be higher in patients with OSAHS compared to healthy subjects during wakeful hours [2,14]. At the onset of sleep, there is a general decrease in muscle tone (hypotonia). This causes a slight increase in airway resistance in healthy subjects, whereas it causes a significant increase in OSAHS patients, who inherently have a smaller airway volume [14,17,18]. The activation of the pharyngeal dilators is known as the upper airway reflex, and its purpose is to force the upper airway to stay open to counteract the negative airway pressure during inhalation [2]. The upper airway is thought to be at the most significant risk of collapse during the time after expiration and before inspiration (when the pharyngeal dilators receive the signals to contract) [2]. However, for OSAHS patients, several factors cause the central respiratory centers to fail to increase the muscle tone of the pharyngeal dilators before the onset of arousal. These factors include impaired sensory information from the upper airway, decreased cortical arousal threshold, or an increased loop gain in the motor control of ventilatory stability [2,14,15]. The failure of the central respiratory centers to increase muscle tone is further amplified and is more prevalent during rapid eye movement (REM) sleep [3,19].

The site of airway obstruction during apnea events differs, with intra- and inter-subject variations and with variations in the obstruction site between sitting and supine positions. However, most studies, irrespective of the technique to localize the occlusion, indicate that the primary site of occlusion is the oropharynx, with extension to the laryngopharynx [2,5,14,15,20].

Different imaging methods have been used to locate occlusion sites during OSAHS events. These include MRI imaging, computerized tomography (CT) scanning, fluoroscopy, endoscopy, or acoustic reflectometry [20,21]. Although helpful in localizing the occlusion site and the degree of occlusion, these methods have problematic methodologies, ranging from invasiveness and sleep disruption to excessive radiation exposure [20]. In addition, the cost of these methods prohibits the repetition of these tests, and their complexity limits the ability to perform them at home. This, in turn, impedes efforts to have large-scale screening and diagnosis of OSAHS.

In vivo ultrasonic measurements have been used as a guidance tool in the invasive treatment of OSAHS [22,23]. The imaging capability of ultrasound has been used as a diagnostic tool. Ultrasonic measurements carry the intrinsic feature of being safe, since they are non-invasive and use non-ionizing radiation, which does not require a contrasting agent. It is a relatively inexpensive, uncomplicated, and commonly portable modality [24,25,26]. A-mode telemetry (echo-ranging) is the most straightforward application of ultrasonic telemetry. An acoustic signal is produced using a piezoelectric transducer, vibrating in the medium-to-high radio frequency range (500 kHz–10 MHz). None of the abovementioned studies have suggested the use of ultrasonic measurement during a full night sleep to capture the presence, duration, and degree of closure of the airway during obstructive sleep apnea events, mainly based on A-mode telemetry

In [27,28], the authors have presented a study of the feasibility of non-invasive detection of obstructive sleep apnea/hypopnea events during sleep using ultrasonic sensors. The hypothesis was that an ultrasonic signal transmitted through or reflected from an open airway would have different features than those associated with a completely or partially occluded airway. To test this hypothesis, the authors constructed an in vitro phantom model of the upper airway that approximates the airway’s and neck’s anatomical dimensions and acoustic properties. It allowed the in vitro simulation of partial and complete occlusion of the upper airway, analogous to that occurring during apnea and hypopnea events associated with OSAHS. The results of the established study confirmed the stated hypothesis.

In this paper, the authors aim to present the experimental design, investigation, and hypothesis testing for using ultrasound to detect the presence of airway occlusion in a full night sleep study for patients diagnosed with obstructive sleep apnea/hypopnea.

## 2. Materials and Methods

This section describes the detailed steps taken to meet the objectives of this study. We have recruited nine patients previously diagnosed with OSAHS. They underwent full night NPSG along with being fitted with the ultrasonic sensory. Data from both NPSG and ultrasound stations were synched and simultaneously collected. The signals were then processed and further analyzed.

### 2.1. Patient Recruitment

The local IRB approved the protocol for patient recruitment and testing and the consent form. The target group of patients was those previously diagnosed with OSA or those highly suspected of having SDB, as referred by the sleep lab (Sleep Consultants, Inc., Fort Worth, TX, USA), who were collaborators in this study.

#### 2.1.1. Inclusion Criteria

The patients could be considered for inclusion in the study if they had previously been diagnosed or were suspected of having OSAHS, as referred by a sleep specialist. There are no restrictions on gender, ethnicity, or body mass index. Recruited patients had to be 21 years of age or older.

#### 2.1.2. Exclusion Criteria

Patients were excluded from the study if they had:

- Known or been diagnosed to have a sensitivity to ultrasonic gel or mineral oil (baby oil);
- Known or been diagnosed to have abnormal growth or tumor in or near the upper airway;
- Difficulty with swallowing;
- Known or been diagnosed to have insomnia;
- Known or suspected of having neurological disorders that may mediate SDB;
- Known or suspected of having cardiovascular conditions that may increase patient risk during the study.

### 2.2. Patient Demography

Twelve patients were recruited for the study. However, only nine patients’ data were used. Three patients’ data were withdrawn from the study due to underlying, previously undisclosed respiratory disorder (asthma) or signal record corruption. Table 1 summarizes the demographics of the nine patients included in the study and the NPSG study findings.

The BMI is calculated from the patient’s weight, W, in kg, and height, H, in meters as BMI = W/H^2^. The total sleep period (TSP) is the time from lights off to lights on during the night study. The total sleep time (TST) is the actual time the patient was asleep. The TSP and TST are based on scoring the 30-s epochs according to the Rechtshaffen and Kales scoring method. Sleep efficiency is the ratio of TST to TSP. The respiratory event indices are calculated as the total number of events divided by the TST, where the units are event/h.

If the patient had already been receiving treatment via the continuous positive airway pressure (CPAP) machine, they were requested to sleep at least two nights without the CPAP before the scheduled study night. It has been shown that apneas generally reappear in patients with OSAHS when they interrupt their CPAP treatment. Therefore, the request for at least a two-night CPAP interruption was required, since the severity of apneas of a single-night interruption was less than the pre-treatment levels [29].

### 2.3. Full Night NPSG Sleep Study

To validate the presence airway occlusions, the patients were concurrently monitored using full-night NPSG. The biosensors used for NPSG were: 4-channel electroencephalography (EEG), left and right electrooculography (EOG), chin and leg electromyography (EMG), electrocardiography (ECG), snoring, nasal airflow, chest and abdominal movement, and arterial oxygen saturation (SaO_2_).

NPSG remains the gold standard in the field of sleep medicine. Sleep medicine physiologists rely on expert scoring of the NPSG by a sleep technologist to identify the sleep architecture, quality of sleep, and different sleep-related disorders. A sleep technologist blind to the objectives of the study scored the NPSG data to determine the apnea, hypopnea, and other respiratory events during sleep. For our research purposes, a synchronizing signal was provided into the ultrasonic data acquisition station and the NPSG data acquisition system (Embla Sandman, Natus Neurology, Inc., Middleton, WI, USA).

### 2.4. Experimental Setup

**Figure 1:**
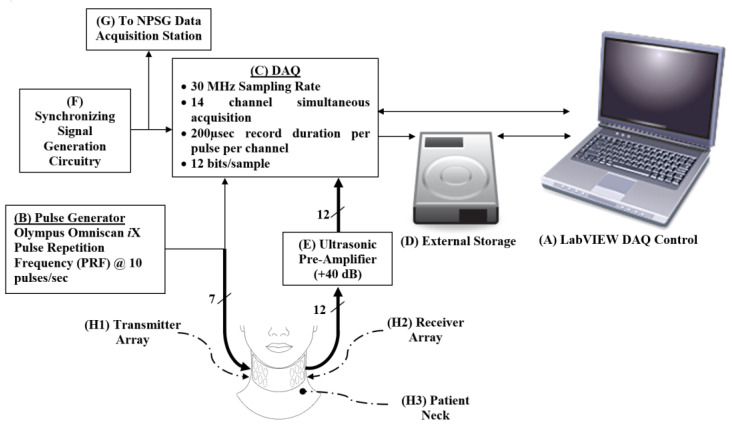
Schematic showing the hierarchy, control, and storage of the signals collected from the phantom. An Omniscan iX (**B**) generates pulses that are transmitted via a transducer array (**H1**) to the patient’s neck (**H3**) and is collected by the receiver array (**H2**) on the opposite side of the neck. The received signal is amplified using the pre-amplifier (**E**). The DAQ (**C**) collects signals and is controlled by a LabVIEW program on the laptop (**A**). The onset of sampling is triggered by the function generator (**F**), which is also used to synchronize the other data collection stations during the night study (**G**). The collected data are streamed and stored in the external high-speed hard drive (**D**).

The main experimental setup used for this study was developed and used in the in vitro study described in [27]. The main components of the experimental setup were the (1) ultrasonic transmitter and receiver arrays, (2) pulse generator, (3) ultrasonic pre-amplifier, and (4) data acquisition station. Figure 1 illustrates the schematic block diagram of the experimental setup.

#### 2.4.1. Transmitter and Receiver Arrays

In designing and fabricating the sensors for detecting the presence of an occlusion in the upper airway during apneic episodes, the following design consideration needed to be addressed [30]:

- Considering the neck curvature, the sensors must have a small surface while being large enough for handling and assembly;
- The curvature of the neck also requires the receivers’ ability to detect the ultrasonic pulses at either normal or oblique incidence;
- The heterogeneity of the tissue media (i.e., skin, fat, muscle, bone, cartilage, air, etc.);
- The main site of occlusion differs between patients, which requires a longitudinal array of sensors, pulsed simultaneously, to capture the occlusion when it occurs and to allow reception spatial resolution to estimate the location of the occlusion.
- The housing of the sensor arrays must be flexible to adhere to the neck curvature, allowing complete contact between the sensors and the skin;
- The sensor array housing must allow for a full night study, which includes the patient’s movement and avoids repeated application of the acoustic gel.

For the transmitter sensors (item (H1) in Figure 1), and to achieve maximum acoustic energy penetration through the neck, a low frequency and large surface area sensor is ideal [25]. However, optimization between those two requirements is required, since the thickness of a piezoelectric sensor is equal to half the wavelength, and a low-frequency sensor doubles in thickness as the frequency is decreased by one-half [25]. In addition, it would be difficult for a sensor with a large surface area to maintain complete skin contact during the full night sleep study on the neck curvature.

**Figure 2:**
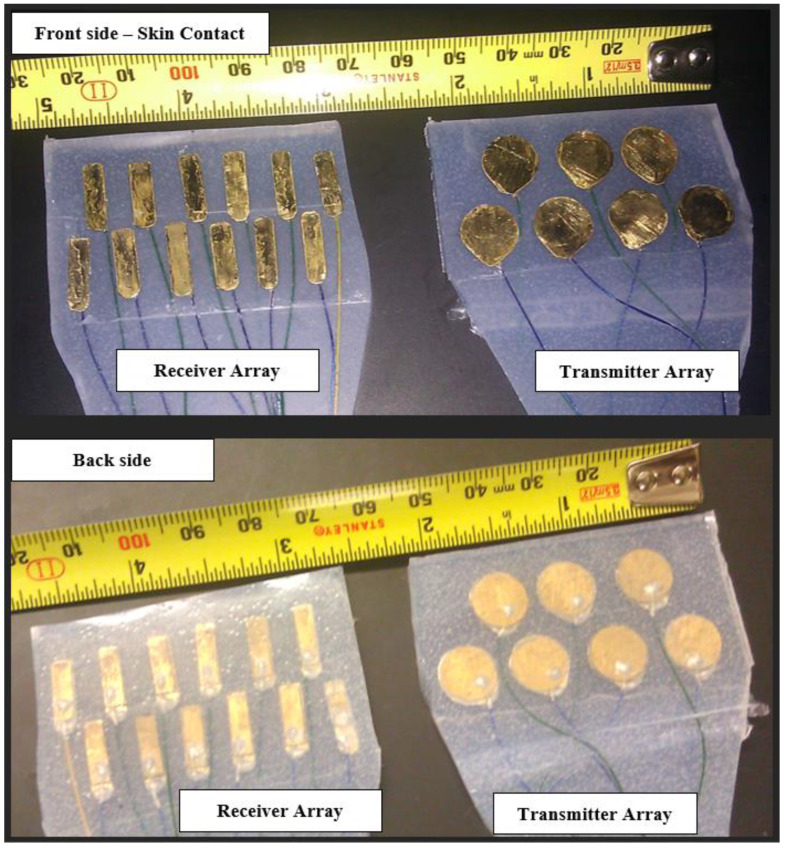
The transmitter and receiver arrays were fabricated for this study. Gold-plated PZT elements were soldered to the brass layer using silver epoxy. The cable electrodes were cold welded to the transducer plates using silver epoxy. The cable is a medical-grade 20-cable shielded ribbon coax cable, each coax having a 0.08 mm diameter [AWG 40] (Hitachi Cable Manchester, Manchester, New Hampshire, USA). The elements were placed within a flexible silicone rubber mold.

To address these practical considerations and limitations for the transmitter array, we designed and fabricated a 7-element double array with low-Q (damped) PZT-5A gold-plated circular piezoelectric transducers with a resonant central frequency of 3MHz (thickness 0.58 mm) and a radius of a = 5 mm, as shown in Figure 2.

These elements were contained in a custom-made silicone rubber mold that allowed for sufficient flexibility and durability for repeated full-night studies for different patients. Acoustic damping was achieved via a brass (Cu_0.7_–Zn_0.3_) backing layer, designed to create the desired Gaussian envelope of the pressure wave. The total thickness of the assembly was less than 2.5 mm.

**Figure 3:**
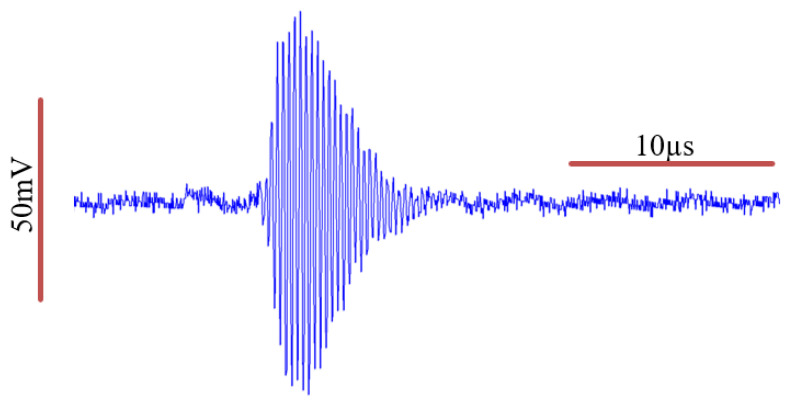
Transmitter element characterization of the acoustic pressure wave in a water tank. An estimated Gaussian envelope of the wave can be identified.

We examined the time domain characteristics of the pulsed mode operation of the transmitter elements in a water tank, which are illustrated in Figure 3.

**Figure 4:**
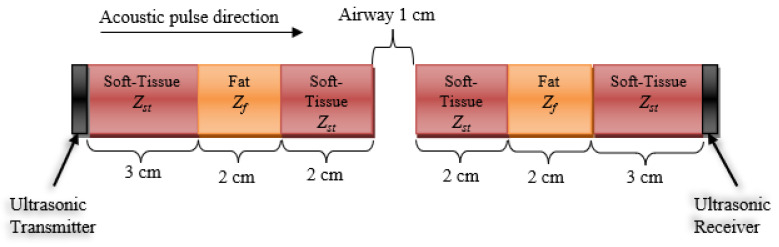
A stratified model of a normal-sized neck and its tissue composition with an open airway in the path of an acoustic pulse traveling left to right (from a transducer on the neck surface, through stratified layers of soft tissue and fat, with an acoustic impedance of *Z_st_* and *Z_f_*, respectively. The acoustic impedance of air is *Z_a_*, while it is *Z_t_* for the transducer. The signal loses strength due to reflection and absorption attenuation.

We further examined the frequency domain characteristics of the wave before and after it passes through the soft tissue of the neck, as illustrated in Figure 4. We calculated the Q-value of the transmitted signal to be 18.4, given that the measured full-width half-max (FWHM) bandwidth (−6 dB BW) is 0.17 MHz, and the actual central frequency of *f_c_* is 3.13 MHz.

Hence, we can model the transmitted pulsed wave equation as follows (assuming the pulse is Gaussian-modulated) [31]:

(1) $$s\left(x,t\right)=A_{o}\cdot e^{\alpha_{a}\left(x,f\right)}\cdot \cos\left(2\pi \left(3.13\times 10^{6}\right)\left(t-\frac{x}{1509.1}\right)\right)\cdot e^{-{\left(1.19\times 10^{6}\right)}^{2}\frac{{\left(t-\frac{x}{1509.1}\right)}^{2}}{2}}$$

where *A_o_* is the initial amplitude of the pressure wave; *t* is the time in seconds; *f* is frequency in Hz, *α_a_* is the attenuation coefficient, which is dependent on the media, distance traveled, and frequency component of the pulse; and *x* is the location (in meters) along the *x*-axis at which it is perpendicular to the surface of the transducer.

An analysis of the derivation of this model can be found in [27] and further detailed herein. The attenuation coefficient is a function of the tissue itself and increases linearly or quadratically with a frequency depending on the tissue and the frequency range [25].

To approximate the pressure wave attenuation in the neck, it was noted that the signal strength attenuated as it passed through various media. The absorption loss is frequency-dependent, and as the signal travels through a medium, it undergoes a frequency distribution shift to the left, due to heavier attenuation at higher frequencies, compared to the lower frequencies [32]. We propose the following stratified model of the neck tissues in an attempt to approximate the signal loss at multiple frequencies in the cases of an open airway and an occluded airway.

Case 1: Open Airway

The illustration in Figure 4 represents an estimated stratified model of the lateral cross-section of the neck with an open airway. The soft tissue, fat, air, and transducers’ acoustic impedances are 1.624, 1.404, 0.000422, and 33.7 MRayl, respectively [30]. First, we calculate the loss of the signal due to reflection, which is only dependent on the interfacing mediums. The reflection coefficient for these interfaces can be calculated using the acoustic reflection (*α_r_*) and acoustic transmission (*α_t_*), given as:

(2) $$\alpha_{r}={\left(\frac{Z_{2}-Z_{1}}{Z_{2}+Z_{1}}\right)}^{2}$$

(3) $$\alpha_{r}+\alpha_{t}=1$$

where *Z*_1_ is the acoustic impedance of the medium proximal to the boundary, and *Z*_2_ is the acoustic impedance of the medium distal to the boundary. Additionally, the higher the impedance mismatch between *Z*_1_ and *Z*_2_, the more the pressure wave will be reflected back to the source, and the less it will be transmitted through to the distal medium.

It is noted that there are three types of interfaces: four soft tissue—fat interfaces (ST−F × 4), two soft tissue—air interfaces (ST−A × 2), and two soft tissue—transducer interfaces (ST−T ×2). Hence, *α_r_* and *α_t_* for those three types can be calculated as:

$$\alpha_{a,ST-F}={\left(\frac{Z_{st}-Z_{f}}{Z_{st}+Z_{f}}\right)}^{2}={\left(\frac{1.624-1.404}{1.624+1.404}\right)}^{2}=0.0053\;\to \alpha_{t,ST-F}=1-\alpha_{a,ST-F}=0.9947\;\alpha_{a,ST-A}={\left(\frac{Z_{st}-Z_{a}}{Z_{st}+Z_{a}}\right)}^{2}={\left(\frac{1.624-0.000422}{1.624+0.000422}\right)}^{2}=0.9995\;\to \alpha_{t,ST-A}=1-\alpha_{a,ST-A}=0.0005\;\alpha_{a,ST-T}={\left(\frac{Z_{st}-Z_{t}}{Z_{st}+Z_{t}}\right)}^{2}={\left(\frac{1.624-33.7}{1.624+33.7}\right)}^{2}=0.9081\;\to \alpha_{t,ST-T}=1-\alpha_{a,ST-T}=0.0919$$

Hence, we can calculate for an acoustic wave with a nominal power of *A_o_* to arrive at the receiver side with a power of (0.9947)^4^ (0.0005)^2^ (0.0919)^2^ *A_o_* = 2 × 10^−9^*A_o_*, or around −87 dB loss. Notice that most of the loss is due to the soft tissue—air interface.

To calculate the attenuation due to absorption, we used the absorption attenuation estimation of the signal in the soft tissue of 0.5 dB/cm/MHz [24]. So, for an acoustic wave at 1 MHz traveling through the tissue model in Figure 4, the signal experiences a loss of 0.5 dB/cm/MHz × 14cm = 7 dB at 1MHz.

Furthermore, an acoustic wave at 2 MHz will experience 14 dB attenuation, and at 3 MHz a 21 dB loss, and so on. This frequency-dependent attenuation causes the distribution of the pulse in the frequency domain to shift to the left [25]. The 1 cm open airway adds an extra 1.6 dB/cm/MHz.

Case 2: Occluded Airway

Using the model in Figure 4, we can examine the attenuation of the signal without the open airway. The attenuation due to the reflection of a signal with a nominal power of *A_o_* can be readily found as (0.9947)^4^ (0.0919)^2^ *A_o_* = 8.3 × 10^−3^ *A_o_*, or about −21 dB loss.

The frequency-dependent attenuation due to absorption of the occluded airway is similar to that of the open airway case, without the 1.6 dB/cm/MHz the air contributes.

Having presented this model and calculation, it is worth mentioning that this is an ideal situation model, and one does not expect the entire signal to pass through the airway during the in vivo testing.

**Figure 5:**
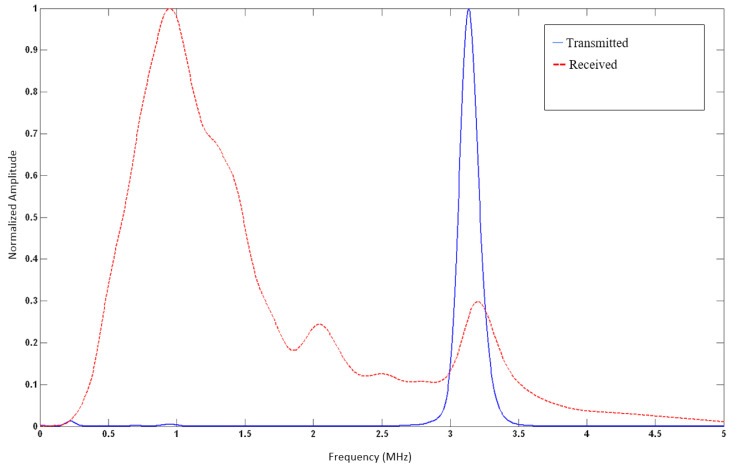
Normalized spectral density characterization of the acoustic pressure wave is seen in Figure 3. Central frequency (*f_c_*) is at 3.13 MHz, with a Q-value of 18.4. An estimated Gaussian envelope of the wave can be readily identified.

This translates to substantial attenuation of the signal’s higher frequency components compared to the lower frequencies as the wave travels in the patient’s neck from the transmitter array to the receiver array for both the open and occluded airway cases. This is illustrated in Figure 5 in the received signal spectra.

For the receiver sensors (subsystem (H2) in Figure 1), a wide divergence angle was desired to allow for the reception of oblique and normal incidence of pressure waves. This can be achieved with rectangular PZT elements. We designed and fabricated a 12-element double array with low-Q (damped) PZT-5A gold-plated rectangular piezoelectric transducers having a resonant central frequency of 3 MHz (thickness 0.58 mm) with width W = 4 mm (8λ) and height H = 10 mm (20λ), where λ = 0.5 mm is the wavelength of the 3 MHz wave in water, as shown in Figure 2.

As with the transmitter elements, the receiver elements were contained in a custom-made silicone-rubber mold that allowed adequate flexibility for full night studies. Acoustic damping was achieved via a brass-backing layer designed to create the desired Gaussian envelope of the pressure wave. The total thickness of the assembly was less than 2.5 mm.

#### 2.4.2. Pulse Generator

An 8-channel OmniScan^®^ iX UT (Olympus NDT, Quebec, Canada) was the source for the ultrasonic pulses. A single channel, set to –100 V, 500 ns width pulse, was used to drive the whole transmitter array (subsystem (B) in Figure 1). That channel, set to a pulse repetition frequency (PRF) of 10 per second, was also used as a trigger of the data acquisition station.

#### 2.4.3. Acoustic Pre-Amplifier

The collected signals at the receiver array were amplified using a 12-channel acoustic wideband pre-amplifier, with an amplification of +40 dB (subsystem (E) in Figure 1). It was designed and constructed at the UT Southwestern Instrumentation Labs, who were collaborators on this study.

#### 2.4.4. Data Acquisition (DAQ) Station

The data acquisition (DAQ) station (subsystem (C) in Figure 1) was an expanded configuration of the system that was used in [27,32]. It was assembled using two National Instruments NI PXI-5105 (Austin, TX, USA) cards, each having 8-channel simultaneously sampled, 60 × 106 samples/s, 12-bits/Sample data acquisition card. They were used to collect the signals at the 12 rectangular ultrasonic receivers. In addition, the transmitted pulse was also recorded, which acted as a soft trigger for the initiation of the 200 µs acquisition window.

In addition, a synchronization signal was also fed into the DAQ (subsystem (F) in Figure 1). This signal was a unique signal with a timestamp encoding and was updated every 5 s. It was shared between the DAQ and the data acquisition system at the sleep lab (Embla Sandman SD32+, Natus Neurology, Inc., Middleton, WI, USA). The role of this signal was to allow for precise alignment of the DAQ recording and the sleep lab data recording for NPSG study and scoring. Real-time data were streamed and stored in 1 terabyte RAID hard drive (NI-8263) (subsystem (D) in Figure 1). The DAQ was controlled with a NI LabVIEW^®^ GUI software (subsystem (A) in Figure 1).

#### 2.4.5. Practical Considerations

To reduce the system’s susceptibility to environmental and power line noise sources, the channels were equipped with isolating coils to reject low-frequency power line 60 Hz interference and its harmonics. Furthermore, the entire length of the coaxial cable and their terminal connections between the transducers, the pulse generator, and the DAQ were shielded inside a 5/8″ braided copper sleeve (AlphaWire, Elizabeth, NJ, USA), acting as a common-point grounded EMI shield.

### 2.5. Study Protocol

After patient consent was obtained, the recruited patient was instrumented with the sensors and electrodes necessary for the full-night NPSG study. The ultrasonic transducer arrays were placed on the patient’s neck behind the ramus of the mandible and immediately below the lobulus auriculae (the earlobe) on both sides of the neck. The ultrasound arrays covered an approximate area of 70 mm × 30 mm. All components of the study were non-invasive.

Upon completion of instrumentation, the patient assumed the prone position to start the full night test and was asked by the lab technicians to go to sleep for 6 to 8 h. Throughout the night, the sleep laboratory trained personnel used two-way voice communication and an infrared camera to monitor the patient. The sleep laboratory personnel were able to respond to the subject’s needs right away. The data were collected for the entire sleep period.

Upon completion of the sleep study and before performing the signal analysis on the collected data, a certified sleep technician—blind to the study’s objectives—scored the NPSG data to identify respiratory events.

### 2.6. Post-Processing

**Figure 6:**
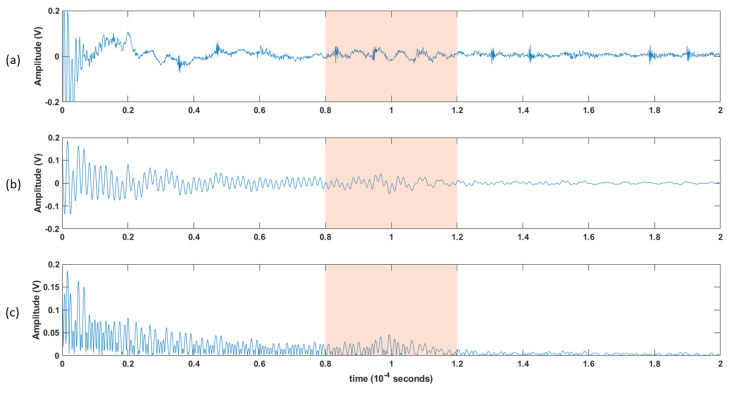
An illustration of the processing steps: (**a**) 200 μs window for the raw collected data. (**b**) the 200 μs collected data after being filtered using a bandpass filter, and then (**c**) rectified. A 40 μs window of interest is shown in the shaded area, and further processing will be applied.

The recorded ultrasonic signals for each patient were stored for post-processing using MATLAB^®^ (Mathworks, Natick, MA, USA). In addition, the sleep-staging and respiratory event annotation from the sleep lab scored data were extracted and synchronized with the ultrasonic data. Figure 6 shows the graphical user interface (GUI) developed for signal post-processing.

**Figure 7:**
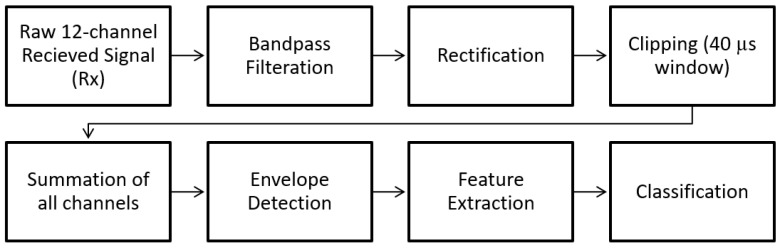
Block diagram showing the post-processing steps.

**Figure 8:**
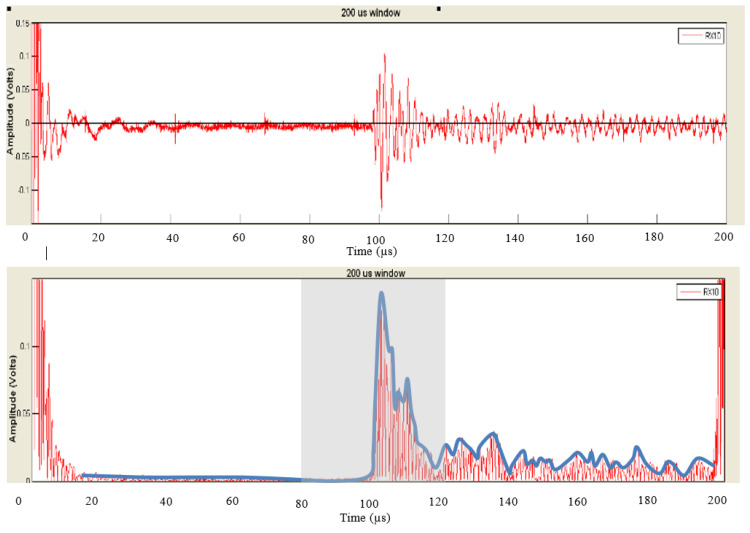
An illustration of the raw signal (**top**) vs. the post-processed result (**bottom**).

The block diagram in Figure 7 shows the steps of post-processing steps, which are as follows (which are further illustrated as the raw versus the post-processed signal waveforms in Figure 8):

1. Each channel was filtered using a Kaiser finite impulse response (FIR) zero-shift bandpass filter, with a lower cutoff frequency of 0.15 MHz and an upper cutoff frequency of 2 MHz. This removed the high-frequency noise, and the baseline was wandering.
2. As the receiver array does not capture any signals until the ultrasonic pulse has traveled the distance representing the diameter of the neck, the first 80 µs (12cm) of the recorded signal does not carry any information. Hence, a 40 µs “window of interest” spanning 80–120 µs was clipped from the 200 µs record. This generalized period represents a signal travel distance of 12–18 cm in the neck. This window corresponds to the expected period of the arrival of the received ultrasonic signal.
3. It is assumed here that changes in the upper airway shape occur at a slower rate than the rate of pulses passing through the airway at 10 pulses per second. Hence, the received signal from every 10 pulses was summed, creating a single waveform per second.
4. Furthermore, the waveforms collected from the channels were added together, resulting in one waveform per second.
5. A cubic spline, sampled at a rate of 30 MHz, was used to estimate the envelope of the resultant waveform connecting the peaks of the resulting signal from the previous step.
6. The envelope was the basis from which temporal and spectral features were extracted, as discussed in the following section.

### 2.7. Feature Extraction

Nine temporal features and four spectral features were extracted from the 40 µs waveform envelopes, resulting from the post-processing steps. These features characterize the envelope’s shape, energy, and spatial and spectral variations. For the spectral features, we calculated the power spectral density of the envelope and identified frequency bands to calculate the area under the spectra.

This resulted in a feature vector of 13 parameters per each second of recording. The extraction of these signal envelope features is summarized in Table 2.

### 2.8. Respiratory Event Epoch Clipping

The patients diagnosed with OSAHS underwent several respiratory episodes during their sleep. First, there was a partial or complete collapse in the airway, causing the OSAHS event, followed by sleep arousal and opening (patency) in the airway for the resumption of breathing. This period is called inter-apneic hyperventilation breathing (HV).

Consequently, to investigate the changes between the respiratory events (RE), both apneic and hypopnea breathing, as well as the inter-apneic HV, we clipped the patient data during the night based on their respective NPSG scores, as assigned by the sleep specialist. Furthermore, since each RE event was followed by a period of breathing resumption, we proposed comparing these events (irrespective of their duration) to the subsequent period of at least 10 s of resumed breathing in which the airway is open.

We considered apneas and mixed apneas as apneic respiratory events (ARE), whereas hypopneas were considered hypopnea respiratory events (HRE). We paired these events with succeeding breathing if and only if the resumed breathing duration was ten seconds or longer. Events followed by less than 10 s of breathing were excluded from being considered in the analysis. Each ARE:HV or HRE:HV pair is called an “event epoch”. Combining ARE:HV and HRE:HV represents all obstructive events and is described hereafter as RE:HV event. Table 3 presents a summary of all event epochs that met the abovementioned criteria, pooled from all nine patient participants in the study.

### 2.9. Hypothesis Formulation

The primary hypothesis of this study is that an ultrasonic signal transmitted through an open (patent) airway will have different temporal and spectral features compared to those associated with a partially or completely occluded airway.

**Figure 9:**
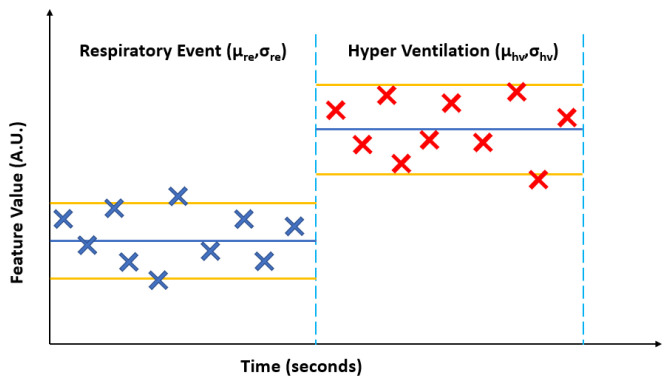
A generalized illustration of calculating the mean (µ) and standard deviation (σ) for an extracted feature from the envelope of the ultrasonic pulses for an apneic or hypopnea event (ARE or HRE, surmised as a respirator event RE) and the succeeding hyperventilation (HV).

To test this hypothesis, we paired event epochs of apneic breathing (partially or fully occluded airway) with their immediate succeeding hyperventilation (patent airway). Furthermore, the extracted 13 temporal and spectral features representing every second of recording for these event epochs were consolidated by calculating each feature’s mean (µ) and standard deviation (σ) for both the RE and succeeding HV. Figure 9 illustrates this process.

Moreover, it must be shown that these extracted features did not significantly change during normal periods of breathing. So, we identified periods of stored data classified as normal breathing. Then, we clipped 20-s epochs of these periods and paired them into clips of normal breathing of 10-s duration (NBx) with succeeding 10 s of normal breathing (NBy) to quantitatively test changes in the features during normal breathing (patent airway).

Based on the preceding discussion, the stated problem needs to be tackled in two steps:

First, we must show the following:

**Clause** **1.** *There is no significant difference (p > 0.05) in the mean of the extracted features during normal breathing for periods longer than 10 s*.

**Clause** **2.** *There is no significant difference (p > 0.05) in the standard deviation of the extracted features during normal breathing for periods longer than 10 s*.

Second, once the abovementioned statements are correct, we hypothesize that the extracted features’ calculated mean and standard deviation difference between the apneic events and the succeeding hyperventilation. The stated hypothesis can be divided into two parts:

**Hypothesis** **H1.** *There is a significant difference (p < 0.05) between the mean RE and mean HV for feature m for each event epoch. The null hypothesis (H_o_) states no difference between mean RE and mean HV features*.

**Hypothesis** **H2.** *There is a significant difference (p < 0.05) between the standard deviation of RE and the standard deviation of HV for feature m for each event epoch. The null hypothesis (H_o_) states that there is no difference between the standard deviation of RE and a standard deviation of HV features*.

It is worth noting here that we assume that the patient does not change their sleep position during the respiratory event and the subsequent resumption of breathing. Specifically, we assumed no change in the baseline of the received signal during each respiratory epoch.

### 2.10. Statistical Analysis and Hypothesis Testing

To normalize for inter- and intra-subject variability, we calculated the logarithm of the ratio for the features’ statistical parameters listed in Table 4.

Therefore, for a feature *m* in Table 2, where 1 ≤ *m* ≤ 13, we calculate the following for the identified clips in Table 3:

(4) $$\mathrm{Ratio}\;\mathrm{of}\;\mathrm{means}\;\mathrm{for}\;\mathrm{NBx}:\mathrm{NBy}RM_{m}=log_{10}\left(\frac{\mu_{m,k}^{NBy}}{\mu_{m,k}^{NBx}}\right)\;1\;\leq \;k\;\leq \;603\;$$

(5) $$\mathrm{Ratio}\;\mathrm{of}\;\mathrm{StDev}\;\mathrm{for}\;\mathrm{NBx}:\mathrm{NBy}RS_{m}=log_{10}\left(\frac{\sigma_{m,k}^{NBy}}{\sigma_{m,k}^{NBx}}\right)\;1\;\leq \;k\;\leq \;603$$

(6) $$\mathrm{Ratio}\;\mathrm{of}\;\mathrm{means}\;\mathrm{for}\;\mathrm{HV}:\mathrm{ARE}\;RM_{m}=log_{10}\left(\frac{\mu_{m,k}^{HV}}{\mu_{m,k}^{ARE}}\right)\;1\;\leq \;k\;\leq \;693$$

(7) $$\mathrm{Ratio}\;\mathrm{of}\;\mathrm{means}\;\mathrm{for}\;\mathrm{HV}:\mathrm{HRE}RM_{m}=log_{10}\left(\frac{\mu_{m,k}^{HV}}{\mu_{m,k}^{HRE}}\right)\;1\;\leq \;k\;\leq \;801$$

(8) $$\mathrm{Ratio}\;\mathrm{of}\;\mathrm{means}\;\mathrm{for}\;\mathrm{HV}:\mathrm{RE}RM_{m}=log_{10}\left(\frac{\mu_{m,k}^{HV}}{\mu_{m,k}^{RE}}\right)\;1\;\leq \;k\;\leq \;1494$$

(9) $$\mathrm{Ratio}\;\mathrm{of}\;\mathrm{StDev}\;\mathrm{for}\;\mathrm{HV}:\mathrm{ARE}\;RS_{m}=log_{10}\left(\frac{\sigma_{m,k}^{HV}}{\sigma_{m,k}^{ARE}}\right)\;1\;\leq \;k\;\leq \;693$$

(10) $$\mathrm{Ratio}\;\mathrm{of}\;\mathrm{StDev}\;\mathrm{for}\;\mathrm{HV}:\mathrm{HRE}RS_{m}=log_{10}\left(\frac{\sigma_{m,k}^{HV}}{\sigma_{m,k}^{HRE}}\right)\;1\;\leq \;k\;\leq \;801$$

(11) $$\mathrm{Ratio}\;\mathrm{of}\;\mathrm{StDev}\;\mathrm{for}\;\mathrm{HV}:\mathrm{RE}RS_{m}=log_{10}\left(\frac{\sigma_{m,k}^{HV}}{\sigma_{m,k}^{RE}}\right)\;1\;\leq \;k\;\leq \;1494$$

The calculated logarithms of the ratios provide the following advantages:

1. Normalizing each feature for the subsequent breathing period to the preceding respiratory event, allowing for normalized intra- and inter-subject comparisons.
2. Introduction of two-tail null-hypothesis testing: If there are no changes in the calculated features between the preceding and succeeding breathing periods in each clip, then the logarithm of the ratio is expected to be equal to zero. If there are changes, then the logarithm of the ratio will be ≠ 0.

Hence, if Equations (4) and (5) are equal to zero (within a 95% confidence interval), then the extracted features show no significant changes during regular breathing periods, and Clause 1 and Clause 2 are established. Furthermore, the null hypotheses (*H_o_*) is accepted if there is no difference between respiratory event features and hyperventilation features, and Equations (6)–(11) are equal to zero (within a 95% confidence interval). Otherwise, if there is a significant difference (*p* < 0.05) between the respiratory event features on one side and HV features on the other, then the Ho will be rejected, and our stated hypotheses will be accepted.

In order to test the hypotheses, we calculate Equations (4)–(11) above for all clipped epochs in Table 3. Since the number of epochs is larger than 30, we can assume that the mean of these ratios is normally distributed. We find the z-value (unity variance and zero mean Gaussian distributed variate) of these features using the following:

(12) $$z_{m}^{RM}=\sqrt{q}\frac{\bar{RM_{m}}}{\sigma_{m}^{RM}}$$

(13) $$z_{m}^{RS}=\sqrt{q}\frac{\bar{RS_{m}}}{\sigma_{m}^{RS}}$$

where $z_{m}^{RM}$ is the z-value for the *RM* of the *m*th feature, $z_{m}^{RS}$ is the z-value for the *RS* of the *m*th feature, $\bar{RM_{m}}$ and $\sigma_{m}^{RM}$ are the mean and standard deviations of *RM* of the *m*th feature, respectively, $\bar{RS_{m}}$ and $\sigma_{m}^{RS}$ are the mean and standard deviations of *RS* of the *m*th feature, respectively, and *q* is the number of clipped epochs.

In order to reject the null hypothesis for *RM_m_*, we need to show that$\;\left|z_{m}^{RM}\right|>1.96$. Same goes for *RS_m_*, where we need to show that$\;\left|z_{m}^{RS}\right|>1.96$. Note that |z| = 1.96 corresponds to a cumulative probability of 0.05 (i.e., 0.025 at each end of the distribution).

However, since the variances of *RM_m_* and RSm are unknown, an estimate of its variance is obtained by computing the sample standard deviation. Hence, the confidence intervals around the mean of *RM_m_* and *RS_m_* must be calculated using a *t*-distribution.

Therefore, we can find the 95% confidence interval (*CI*) of the mean for each *RM* and *RS* distribution using the following:

(14) $$R_{m}^{CI}=\bar{RM_{m}}\mp \left(t_{q,0.05/2}\times \frac{\sigma_{m}^{RM}}{\sqrt{q}}\right)$$

(15) $$RS_{m}^{CI}=\bar{RS_{m}}\mp \left(t_{q,\;0.05/2}\times \frac{\sigma_{m}^{RS}}{\sqrt{q}}\right)$$

where *q* is the degrees of freedom (number of clips), and *t_q,0.05/2_* is the Student t-test value for an *α* = 0.05 in a 2-tail test.

For Clause 1 and Clause 2, when plotted for all features 1 ≤ *m* ≤ 13, we need to show that the 95% confidence interval does include zero (i.e., *p*-value > 0.05), so that we can establish that for all features, *m*, there are no significant differences between the epochs containing normal breathing.

Once that is shown, we can investigate Hypotheses *H1* and *H2* by plotting for all features 1 ≤ *m* ≤ 13. If the 95% confidence interval includes zero for feature *m*, then we cannot reject the null hypothesis. This is because the specific feature *m* does not show a significant difference between the apneic event and the subsequent hyperventilation. Conversely, a 95% confidence interval that does not include zero for feature m (*p*-value < 0.05) allows us to reject the null hypothesis; that is, there is a significant difference in that specific feature between the apneic event and the subsequent hyperventilation.

## 3. Results

**Figure 10:**
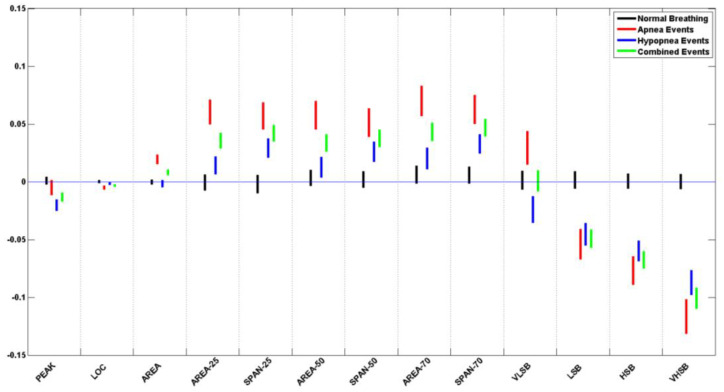
Ratio of means (RM) CI plots for all 13 features of the pooled data from all subjects.

**Figure 11:**
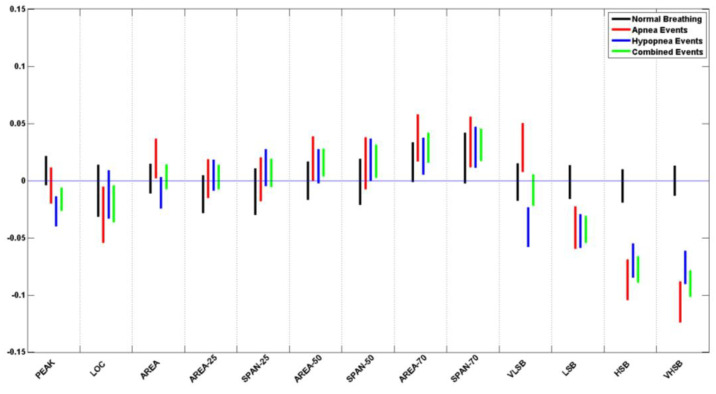
Ratio of standard deviation (RS) CI plots for all 13 features of the pooled data from all subjects.

Table 5 lists the *p*-value for each of the logarithms of the ratio of the means and standard deviation of the extracted features from normal breathing epochs of this study. Figure 10 and Figure 11 contain the illustration of these results.

It can be seen that all of the extracted features do not show a significant difference during normal breathing periods, where all *p*-values > 0.05. When plotted, all the feature confidence intervals contain the value 0.

Table 6 and Table 7 list the *p*-value for each of the logarithms of the ratio of the means and standard deviation of the extracted features from apneic and hypopnea breathing, respectively. Since sleep physicians usually consider the cardiovascular response of apnea and hypopnea in patients to be the same, we have listed the combination of all apneic and hypopnea breathing in Table 8. It includes the list of the *p*-values for each of the logarithms of the ratio of the means and standard deviation of the extracted features. Figure 10 and Figure 11 contain the illustration of these results.

It can be seen that most of the extracted features do show a significant difference between the apneic breathing and the subsequent hyperventilation periods, wherewith *p*-values < 0.05, and the confidence intervals do not contain the value 0. This is particularly evident in the ratio of means more than the ratio of standard deviations. The spectral features LSB, HSB, and VHSB showed the most significant difference between apneic breathing and hyperventilation. In most cases, apnea epochs showed a higher difference than hypopnea epochs; i.e., the CI was further away from the zero line for apnea (ARE) than it was for hypopnea (HRE) for that feature. As expected, combining apnea and hypopnea epochs (RE) in one category results in CI between ARE and HRE confidence intervals.

It must be noted that, unlike the resultant features’ trends, the VLSB feature has CI for apnea events higher than the zero line. In contrast, it is lower than the zero line for hypopnea events for both ratios of means and ratio of standard deviations.

## 4. Discussion

By reviewing the recruited subject demographics and sleep study data in Table 1, It is noticed that the group BMI is equal to 37.6 ± 6.6 kg/m^2^. This falls within the characteristic of the large-scale OSAH population demographics in which obesity is highly correlated with OSAH incidences. Moreover, the group’s AHI of 78.6 ± 39.2 h^−1^ (range 29.3–159.6 h^−1^) is an indicator of the severity of the OSAH classification of the group in which an AHI of 30 h^−1^ or higher is generally considered severe OSAH. This is further illustrated by the lowered TST blood oxygenation of the whole group (SaO_2_ = 93.4% ± 3.3%).

It is noted that the group’s sleep efficiency is low (about 70%), marked by repetitive awakening during the night, mainly due to cortical arousal following respiratory events. Once again, this is in line with the characteristics of poor sleep quality for the OSAH general population, even when provided with a long sleep period (more than 6 h, on average).

In the plotted in situ spectra of the pulsed and detected signal after it travels in the neck, a notable shift-to-the-left effect is due to frequency-dependent attenuation as the ultrasonic pulse passes through the various soft tissues in the neck. The original pulse has a Gaussian distribution and is centered at 3.13 MHz. As it travels through the soft tissue of the neck, it experiences a linearly increasing frequency-dependent absorption, causing the higher frequencies in the pulse to be absorbed at a much higher rate than at lower frequencies. As a result, the received signal has a wideband distribution, with a peak frequency of around 0.9 MHz.

In conducting the in situ for this study, we experimented with different locations to place the ultrasonic array to provide the highest reception sensitivity. We found that placing the ultrasonic on the patient’s neck behind the ramus of the mandible, and immediately below the ear lobe, on both sides of the neck, was the optimum location of the transmission mode.

The mounting of the ultrasonic arrays on the patient’s neck and keeping them in full contact with the skin during the night study was of high importance. We addressed this by designing the in situ transducers that were small in size and without metallic casing and arranging them into two arrays of transmitters and receivers to cover a large area of the two sides of the neck without interfering with the ability of the patient to sleep and move. We placed the transducers in flexible silicon rubber substrates, covered them in ultrasonic aqueous gel, and used surgical tape to keep the arrays in full contact with the skin at night.

Doing away with the metallic casing of the ultrasonic transducers was one of the sources of the system’s susceptibility to electromagnetic interference (EMI) and environmental noise. Therefore, we shielded the cabling system with a breaded copper sheath and used ground isolators to reduce EMI. Furthermore, we performed offline de-noising signal filtration to improve the signal-to-noise ratio (SNR) using a bandpass filter.

We have developed temporal and spectral features to perform statistically quantitative testing of the changes in the received ultrasonic waveform during normal breathing versus airway full or partial occlusion.

It was shown that these developed features do not significantly change during regular breathing periods by establishing the assumptions in Clause 1 and Clause 2. This has allowed for hypotheses testing if significant changes exist in these features when the airway is partially or completely collapsed compared to immediate succeeding periods of a patent airway.

All the spectral features and most of the temporal features show a significant difference between apneic breathing and hyperventilation. The level of significance varied between the features. However, the most noteworthy of these features were the spectral features for both the ratio of means and the ratio of standard deviation. These features had the same tendency to be above or below the zero line for apneic events with overlapping confidence intervals, except for the VLSB features. Whereas the significantly different features can be used to distinguish apneic events, the VLSB can be readily used to discern the type of event as apnea or hypopnea, since it has confidence intervals above the zero line for apnea events and below the zero line for hypopnea epochs.

## 5. Conclusions

This work is the first to show that it is feasible to use the ultrasonic signal to detect airway closure due to obstructive sleep apnea. The feasibility has been in situ after this purpose’s development and fabrication of customized ultrasonic arrays. The analysis method presented included temporal and spectral domain features extracted from apneic and normal breathing epochs. Statistical analysis has shown that the ultrasonic pulses passing through the patient’s neck undergo changes that significantly differ between patent airway during normal breathing and partially or completely collapsed airway during apneic breathing. This work opens future avenues for the investigation of stand-alone automated ultrasound devices that can be used for large-scale OSAHS screening. Future work for the proposed ultrasonic detection system includes machine learning and further analysis of the data to derive indices of the severity of apnea from the ultrasound signal, such as the apnea–hypopnea index. Additionally, the possibility of studying other types of sleep apnea (central and mixed) and Cheyne–Stokes syndrome is of interest.

## 6. Patents

A patent application was filed for the work presented in this article: US Patent App. 13/588,819.

## Figures and Tables

**Table 1:** Patient demographics and NPSG study findings.

Variable	Stats
Patients (Number, Gender)	2 Females, 7 Males
Age (years) (mean ± SD)	41.9 ± 10.6
Height (m) (mean ± SD)	1.75 ± 0.14
Weight (kg) (mean ± SD)	114.8 ± 25.5
Neck measurement (cm, coronal) (mean ± SD)	15.2 ± 1.1
BMI (kg/m^2^) (mean ± SD)	37.6 ± 6.6
Total Sleep Period (min) (mean ± SD)	368.7 ± 75.7
Total Sleep Time (min) (mean ± SD)	270.3 ± 68.3
Sleep Efficiency Index (%) (mean ± SD)	70.9% ± 13.4%
Total Sleep Time Mean SaO_2_ (%) (mean ± SD)	93.4% ± 3.3%
Obstructive Apnea Index (mean ± SD)	29.2 ± 26.9
Hypopnea Index (mean ± SD)	41.7 ± 30.3
Mixed Apnea Index (mean ± SD)	5.5 ± 9.8
Central Apnea Index (mean ± SD)	1.0 ± 1.65
Apnea/Hypopnea Index (AHI) (mean ± SD)	78.6 ± 39.2

**Table 2:** List of temporal and spectral features extracted from ultrasonic envelopes.

Feature Index (*m*)	Feature	Description	Classification
1	PEAK	Maximum value of the envelope	Temporal
2	LOC	Temporal location of peak	Temporal
3	AREA	Total area under the curve	Temporal
4	AREA-25	Area under the curve bounded by the first crossing of the 25% threshold to left and right of peak	Temporal
5	SPAN-25	Duration of the waveform between the first crossing of the 25% of peak threshold to left and right of peak	Temporal
6	AREA-50	The area under the curve bounded by the first crossing of the 50% threshold to left and right of peak	Temporal
7	SPAN-50	Duration of the waveform between the first crossing of the 50% of peak threshold to left and right of peak	Temporal
8	AREA-70	The area under the curve bounded by the first crossing of the 70% threshold to left and right of peak	Temporal
9	SPAN-70	Duration of the waveform between the first crossing of the 70% of peak threshold to left and right of peak	Temporal
10	VLSB	Very Low Spectral Band (10–230 kHz)	Spectral
11	LSB	Low Spectral Band (230–487 kHz)	Spectral
12	HSB	High Spectral Band (487–770 kHz)	Spectral
13	VHSB	Very High Spectral Band (770–1000 kHz)	Spectral

**Table 3:** Total number and classification of event epochs clipped from all participants in the study.

Event Epoch Classification	Number of Clipped Epochs
Control normal breathing of 10 s followed by normal breathing for 10 s (NB:NB)	603
Apneic respiratory event followed by at least 10 s of hyperventilation (ARE:HV)	693
Hypopnea respiratory event followed by at least 10 s of hyperventilation (HRE:HV)	801
Combine respiratory event (apnea and hypopnea) followed by at least 10 s of hyperventilation (RE:HV) = (ARE:HV) + (HRE:HV)	1494

**Table 4:** List of statistical parameters.

Statistical Parameter	Description
$\mu_{m}^{ARE}$	Feature *m* mean for apneic respiratory event (*ARE*)
$\mu_{m}^{HRE}$	Feature *m* mean for hypopnea respiratory event (*HRE*)
$\mu_{m}^{RE}$	Feature *m* mean for all respiratory events (*RE*)
$\mu_{m}^{HV}$	Feature *m* mean for succeeding hyperventilation period (*HV*)
$\mu_{m}^{NBx}$	Feature *m* mean for the first 10 s controlling normal breathing (*NBx*)
$\mu_{m}^{NBy}$	Feature *m* mean for the second 10 s controlling normal breathing (*NBy*)
$\sigma_{m}^{ARE}$	Feature *m* standard deviation for apneic respiratory event (*ARE*)
$\sigma_{m}^{HRE}$	Feature *m* standard deviation for hypopnea respiratory event (*HRE*)
$\sigma_{m}^{RE}$	Feature *m* standard deviation for all respiratory events (*RE*)
$\sigma_{m}^{HV}$	Feature *m* standard deviation for succeeding hyperventilation period (*HV*)
$\sigma_{m}^{NBx}$	Feature *m* standard deviation for the first 10 s controlling normal breathing (*NBx*)
$\sigma_{m}^{NBy}$	Feature *m* standard deviation for the second 10 s controlling normal breathing (*NBy*)

**Table 5:** List of temporal and spectral features extracted from ultrasonic envelopes for normal breathing (*N* = 603).

Feature Index (*m*)	Feature	Ratio of Means (RM)	Ratio of Standard Deviations (RS)
		*p*-Value	*p*-Value
1	PEAK	0.527	0.163
2	LOC	0.946	0.443
3	AREA	0.731	0.781
4	AREA-25	0.857	0.160
5	SPAN-25	0.621	0.353
6	AREA-50	0.339	0.994
7	SPAN-50	0.551	0.929
8	AREA-70	0.111	0.067
9	SPAN-70	0.123	0.074
10	VLSB	0.757	0.877
11	LSB	0.657	0.863
12	HSB	0.831	0.520
13	VHSB	0.949	0.998

**Table 6:** Apnea hypothesis testing (bold for *p*-value < 0.05, *N* = 693).

Feature Index (*m*)	Feature	Ratio of Means (RM)	Ratio of Standard Deviations (RS)
		*p*-Value	*p*-Value
1	PEAK	0.117	0.591
2	LOC	**<0.001**	**0.017**
3	AREA	**<0.001**	**0.027**
4	AREA-25	**<0.001**	0.834
5	SPAN-25	**<0.001**	0.895
6	AREA-50	**<0.001**	0.052
7	SPAN-50	**<0.001**	0.188
8	AREA-70	**<0.001**	**<0.001**
9	SPAN-70	**<0.001**	**0.002**
10	VLSB	**<0.001**	**0.008**
11	LSB	**<0.001**	**<0.001**
12	HSB	**<0.001**	**<0.001**
13	VHSB	**<0.001**	**<0.001**

**Table 7:** Hypopnea hypothesis testing (bold for *p*-value < 0.05, *N* = 801).

Feature Index (*m*)	Feature	Ratio of Means (RM)	Ratio of Standard Deviations (RS)
		*p-*Value	*p-*Value
1	PEAK	**<0.001**	**<0.001**
2	LOC	**0.014**	0.258
3	AREA	0.325	0.126
4	AREA-25	**<0.001**	0.496
5	SPAN-25	**<0.001**	0.161
6	AREA-50	**0.004**	0.094
7	SPAN-50	**<0.001**	0.050
8	AREA-70	**<0.001**	**0.009**
9	SPAN-70	**<0.001**	**0.001**
10	VLSB	**<0.001**	**<0.001**
11	LSB	**<0.001**	**<0.001**
12	HSB	**<0.001**	**<0.001**
13	VHSB	**<0.001**	**<0.001**

**Table 8:** Combined hypothesis testing (bold for p-value < 0.05, *N* = 1494).

Feature Index (*m*)	Feature	Ratio of Means (RM)	Ratio of Standard Deviations (RS)
		*p-*Value	*p-*Value
1	PEAK	**<0.001**	**0.001**
2	LOC	**<0.001**	**0.012**
3	AREA	**<0.001**	0.544
4	AREA-25	**<0.001**	0.538
5	SPAN-25	**<0.001**	0.284
6	AREA-50	**<0.001**	**0.010**
7	SPAN-50	**<0.001**	**0.021**
8	AREA-70	**<0.001**	**<0.001**
9	SPAN-70	**<0.001**	**<0.001**
10	VLSB	0.864	0.231
11	LSB	**<0.001**	**<0.001**
12	HSB	**<0.001**	**<0.001**
13	VHSB	**<0.001**	**<0.001**

## Data Availability

Not applicable.

## References

[1] WHO (2016). International Statistical Classification of Diseases and Related Health Problems, 10th Revision, Fifth Edition. https://apps.who.int/iris/handle/10665/246208.

[2] Broderick M., Guilleminault C. (2008). Neurological aspects of Obstructive Sleep Apnea. Ann. N. Y. Acad. Sci..

[3] Eckert D.J., Malhotra A. (2008). Pathophysiology of adult Obstructive Sleep Apnea. Proc. Am. Thorac. Soc..

[4] Sateia M.J. (2014). International classification of sleep disorders. Chest.

[5] Fogel R.B., Malhotra A., White D.P. (2004). Sleep 2: Pathophysiology of Obstructive Sleep Apnoea/Hypopnoea syndrome. Thorax.

[6] Carter R., Watenpaugh D.E. (2008). Obesity and obstructive sleep apnea: Or is it OSA and obesity?. Pathophysiology.

[7] Peppard P.E., Young T., Palta M., Dempsey J., Skatrud J. (2000). Longitudinal study of moderate weight change and sleep-disordered breathing. J. Am. Med. Assoc..

[8] Punjabi N.M. (2008). The epidemiology of adult Obstructive Sleep Apnea. Proc. Am. Thorac. Soc..

[9] Young T., Peppard P.E., Gottlieb D.J. (2002). Epidemiology of Obstructive Sleep Apnea: A population health perspective. Am. J. Respir. Crit. Care Med..

[10] Edwards B.A., Wellman A., Owens R.L. (2013). Psgs: More than just the ahi. J. Clin. Sleep. Med..

[11] Peppard P.E., Young T., Barnet J.H., Palta M., Hagen E.W., Hla K.M. (2013). Increased prevalence of sleep-disordered breathing in adults. Am. J. Epidemiol..

[12] Flemons W.W., Douglas N.J., Kuna S.T., Rodenstein D.O., Wheatley J. (2004). Access to diagnosis and treatment of patients with suspected sleep apnea. Am. J. Respir. Crit. Care Med..

[13] Young T., Palta M., Dempsey J., Peppard P.E., Nieto F.J., Hla K.M. (2009). Burden of Sleep Apnea: Rationale, design, and major findings of the Wisconsin Sleep Cohort Study. Wis. Med. J..

[14] White D.P. (2006). Sleep Apnea. Proc. Am. Thoraic Soc..

[15] Agur A.M.R., Lee M.J., Kelly P.J. (1999). Grant’s Atlas of Anatomy.

[16] Rodenstein D.O., Dooms G., Thomas Y., Liistro G., Stanescu D.C., Culee C., Aubert-Tulkens G. (1990). Pharyngeal shape and dimensions in healthy subjects, snorers, and patients with Obstructive Sleep Apnoea. Thorax.

[17] Schwab R.J., Pasirstein M., Pierson R., Mackley A., Hachadoorian R., Arens R., Maislin G., Pack A.I. (2003). Identification of upper airway anatomic risk factors for Obstructive Sleep Apnea with volumetric magnetic resonance imaging. Am. J. Respir. Crit. Care Med..

[18] Martins A.B., Tufik S., Moura S.M.G.P.T. (2007). Physiopathology of Obstructive Sleep Apnea-Hypopnea Syndrome. J. Bras. De Pneumol..

[19] Rama A.N., Tekwani S.H., Kushida C.A. (2002). Sites of obstruction in Obstructive Sleep Apnea. Chest.

[20] Faber C.E., Grymer L. (2003). Available techniques for objective assessment of upper airway narrowing in snoring and Sleep Apnea. Sleep Breath..

[21] Maturo S.C., Mair E.A. (2006). Submucosal minimally invasive lingual excision: An effective, novel surgery for pediatric tongue base reduction. Ann. Otol. Rhinol. Laryngol..

[22] Robinson S., Lewis R., Norton A., McPeake S. (2003). Ultrasound-guided radiofrequency submucosal tongue-base excision for Sleep Apnoea: A preliminary report. Clin. Otolaryngol. Allied Sci..

[23] Quistgaard J. (1997). Signal acquisition and processing in medical diagnostic ultrasound. IEEE Signal Process. Mag..

[24] Hedrick W.R., Hykes D.L., Starchman D.E. (2005). Ultrasound Physics and Instrumentation.

[25] Cobbold R.S. (2007). Foundations of Biomedical Ultrasound.

[26] Al-Abed M., Antich P., Watenpaugh D.E., Behbehani K.E. Detection of airway occlusion in simulated Obstructive Sleep Apnea/Hypopnea using ultrasound: An in vitro study. Proceedings of the 32nd Annual International Conference of the IEEE Engineering in Medicine and Biology Society.

[27] Al-Abed M., Behbehani K., Antich P., Watenpaugh D., Burk J. (2013). Systems and Methods for Detecting Airway Occlusion. U.S. Patent.

[28] Boudewyns A., Sforza E., Zamagni M., Krieger J. (1996). Respiratory effort during Sleep Apneas after interruption of long-term Cpap treatment in patients with Obstructive Sleep Apnea. Chest.

[29] Al-Abed M., Antich P., Watenpaugh D.E., Behbehani K. In Vivo characterization of ultrasonic sensors for the detection of airway occlusion in sleep disordered breathing. Proceedings of the 33rd Annual International Conference of the IEEE Engineering in Medicine and Biology Society.

[30] Al-Abed M.A. (2011). Non-Invasive Detection of Upper Airway Occlusion Using Piezoelectric Ultrasonic Sensors in Sleep Apnea Patients. Ph.D. Thesis.

[31] Al-Abed M., Antich P., Watenpaugh D.E., Behbehani K. (2017). Phantom study evaluating detection of simulated upper airway occlusion using piezoelectric ultrasound transducers. Comput. Biol. Med..

[32] Al-Abed M.A., Antich P., Watenpaugh D.E., Behbehani K. Upper airway occlusion detection using a novel ultrasound technique. Proceedings of the Engineering in Medicine and Biology Society (EMBC), 2012 Annual International Conference of the IEEE.

